# Prevalence, Pattern, and Predictors of Premenstrual Syndrome (PMS) and Premenstrual Dysphoric Disorder (PMDD) in Asir Region, Saudi Arabia

**DOI:** 10.7759/cureus.65723

**Published:** 2024-07-30

**Authors:** Anna Zarfishan, Saeed Abdullah Saeed Alqahtani, Faris A Alasmre, Hind A Alasmre, Lujain A Alasmre, Raghad A Alarim, Ali A Almuntashiri, Abdullah M Al Shahrani, Rasha Saeed Mohammed Alqahtani

**Affiliations:** 1 Department of Obstetrics and Gynecology, King Khalid University, Abha, SAU; 2 Obstetric and Gynecology, Abha Maternity and Children Hospital, Abha, SAU; 3 Obstetrics and Gynecology, Abha Maternity and Children Hospital, Abha, SAU; 4 College of Medicine and Surgery, King Khalid University, Abha, SAU; 5 Department of Internal Medicine, King Khalid University, Abha, SAU; 6 General Practice, Um Sarar Primary Health Care Center, Khamis Mushait, SAU; 7 General Practice, Abha Maternity and Children Hospital, Abha, SAU

**Keywords:** saudi arabia, asir region, premenstrual dysphoric disorder (pmdd), premenstrual syndrome (pms), predictors, pattern, prevalence

## Abstract

Background: The prevalence and consequences of premenstrual dysphoric disorder (PMDD) and premenstrual syndrome (PMS) among Saudi women are not well understood. Consequently, the purpose of this research is to ascertain the frequency, course, and contributing factors of PMDD and PMS in the Asir area of Saudi Arabia.

Methods: A cross-sectional study was conducted in Asir, Saudi Arabia, using a pre-designed questionnaire distributed among adult female patients to five primary healthcare facilities (PHCs) that were chosen at random in the Asir Region. Data were analyzed using IBM Statistical Package for the Social Sciences (SPSS, version 15).

Results: The study included 506 participants; 81% of them were Saudi and 19% were non-Saudi. A percentage (46.2%) of the participants were aged between 21 and 30 years old. A total of 369 (72.9%) participants have PMS. Regarding PMS symptoms, 79.1% reported mood changes, 59.3% tantrums, 56.7% change in appetite, 47% acne, 41.9% back and joint pain, and 43.1% reported sleep disorders. Almost half (42.1%) of the participants receive treatment to relieve the symptoms of PMS (72% of them receive paracetamol and 6.1% receive nonsteroidal anti-inflammatory drugs (NSAIDs)). Some (6.4%) exercise during the menstrual cycle to relieve symptoms of PMS, while 31% eat healthy foods at the time of period to relieve the symptoms of PMS.

Conclusion: The prevalence of PMS/PMDD was among the reported figures worldwide. There was a significant association between age and the use of contraceptives with symptoms of PMS/PMDD among the studied sample.

## Introduction

Premenstrual disorders consist of a series of premenstrual symptoms starting from mild premenstrual syndrome (PMS) to premenstrual dysphoric disorder (PMDD), which is a more severe form. PMS manifests itself during the monthly cycle's luteal stage and goes away for the rest of the cycle; in addition, it disturbs the psychological, physical, and behavioral symptoms [1]. The prevalence of PMS in health colleges in Dammam, Saudi Arabia, was 46.7% [2]. About 24-32% of women reported moderate to severe PMS and 3-8% have a very severe form of PMS, which is PMDD [3]. PMS is therefore predominant in women of all ages affecting considerable morbidity with apparent damage to interpersonal relationships, work performance, lifestyle, social interactions, emotional well-being, and overall health-related quality of life (HRQoL) [4]. This disorder is particularly common in the younger age groups and therefore represents a significant public health problem [5].

Nearly 90% of women have experienced at least one PMS as defined by the International Classification of Diseases, Tenth Revision (ICD-10) criteria. Epidemiological investigations have assessed that around 75% of reproductive-age females experience some symptoms related to the premenstrual phase of the menstrual cycle [6].

The etiology of PMS is uncertain. Since PMS symptoms occur simultaneously with the hormonal fluctuations of the menstrual cycle, hormonal disproportion like estrogen surplus and progesterone deficiency have been proposed. Symptoms are also associated with serotonin to link as a key etiological factor [7,8]. Women have mood swings due to fluctuations in their luteal stage levels of estrogen. Molecular biology research indicates that the hypothalamus releases norepinephrine in response to a drop in estrogen, and it causes acetylcholine, dopamine, and serotonin levels to drop, which in turn causes despair, exhaustion, and insomnia - all classic symptoms of PMDD and PMS [9]. An Egyptian research found a favorable correlation between PMS and consuming too many sweet-tasting foods. It also showed that there was a substantial correlation between PMS and other variables, such as coffee and junk food consumption, demonstrating the strong correlation that lifestyle variables have with PMS and PMDD [10].

Many other conditions, both medical and psychological, must be ruled out in order to diagnose PMS and PMDD. The presence of three criteria, namely, consistency with PMS symptoms (I), constant occurrence of symptoms only during the luteal phase of the menstrual cycle (II), and detrimental effects on the patient's function and lifestyle (III), confirms the diagnosis. Patients should be instructed to record their premenstrual signs for a continuous month in order to check for cycle-to-cycle fluctuation, especially if the doctor has serious doubts about the diagnosis [11].

Reducing the impact of PMS on everyday activities and relieving its symptoms are the primary goals of treatment. PMS was traditionally treated with pharmacotherapy; however, more recent studies have indicated that combination therapy provides greater advantages [12].

Symptoms of PMS can be effectively treated by combining medications [13], such as non-steroidal anti-inflammatory drugs (NSAIDs), selective serotonin reuptake inhibitors (SSRIs), anxiolytics, gonadotropin-releasing hormone (GnRH) agonists, the medication, and oral contraceptive pills, with nonpharmacological therapies, such as cognitive and behavioral therapies, exercises, massage therapy, light therapy, and dietary and nutritional modifications. The majority of signs of PMS might return after therapy is stopped, with the exception of menopausal and oophorectomy [14]. This study looks at PMS and PMDD incidence, patterns, and determinants in Saudi Arabia's Asir Region.

## Materials and methods

This is a cross-sectional study conducted in the Asir Region of Saudi Arabia, a distinctive locale that provided the backdrop for the study's observations and conclusions. The study transpired over a timeframe spanning two months, commencing on July 1, 2022, and concluding on September 31, 2022.

The study targeted a specific demographic cohort, namely, females aged between 18 and 40 years residing in the Asir Region of Saudi Arabia. This selection criterion ensured a coherent and relevant sample group for the research objectives. The inclusion criteria outlined parameters for participation, emphasizing that women within the specified age range and from diverse educational backgrounds were eligible to take part. In addition, the participants were required to express their willingness to engage in the study, thus ensuring informed and voluntary involvement.

Conversely, the exclusion criteria delineated specific conditions that would disqualify individuals from participating in the study. Factors such as age, pregnancy or lactation, and the presence of certain health conditions like gynecological issues or chronic diseases were identified as grounds for exclusion.

Sample size

The minimum sample size for this study has been decided according to Swinscow, as follows: 

n = Z2 x P x Q/D2

Where: 

n: calculated sample size

Z: the z-value for the selected level of confidence [1- a] = 1.96

P: sn estimated prevalence of having PMTS

Q: [1 - 0.50] = 50%, i.e., 0.50

D: the maximum acceptable error = 0.05.

Thus, the calculated minimum sample size was n = [1.96]2 X 0.50 X 0.50 = 384 / [0.05] 2 = 384 women.

It was increased to 420 by adding 10%, to compensate for nonresponses and incomplete forms.

Method of gathering data

The method used for random sampling was methodical. All adult female participants at five randomly chosen PHCs in the Asir Region provided data. Every second lady who attended the research was questioned after the first respondent was chosen at random in order to ensure that the necessary sample size was achieved. In-person conversations with members of the sample population were used to gather data, and they also completed a questionnaire that helped us find the information we needed to meet the goals of the study.

Tool for gathering data

To gather data, a pre-made questionnaire was employed. There are two primary portions to it. The participants' demographic data include information on their age, marital status, educational attainment, height, weight, and number of children. The second segment will evaluate the regularity of menstruation, the kind and effectiveness of any contraception used, the incidence of PMTS symptoms, and any therapeutic options. Prior to the interview, each participant provided written informed consent.

Preliminary investigation

To determine the tool's viability, applicability, and clarity and to make any necessary modifications, a pilot study involving 10 women was conducted. Women were not allowed to participate in the pilot trial.

Data organization and statistical evaluation

Data analysis and tabulation were performed after using the IBM SPSS Statistics for Windows, Version 21.0 (released 2012, IBM Corp., Armonk, NY). The use of descriptive statistics, such as frequency, and percentage was implemented. P-values were deemed significant if they were less than 0.05, and appropriate analytical tests of significance were employed.

Ethical considerations

The Research Ethics Committee at King Khalid University (HAPO-06-B-001) issued approval (approval no. ECM#2023-3242). The participants had the option to withdraw from the study anytime they felt it was necessary, and confidentiality and anonymity of the data were guaranteed throughout the investigation. A conflict of interest does not exist.

## Results

As illustrated in Table 1, out of 506 participants, 81% were Saudi and 19% were non-Saudi. A total of 234 participants (46.2%) were aged between 21 and 30 years old, 24.5% (124 participants) were less than 20 years old, and 15.6% (79 participants) were in the 31-40 age group. A percentage (22.5%, 114 participants) were overweight, and 17% (86 participants) were obese. Regarding marital status, 57.3% (290 participants) were single, and 41.1% (208 participants) were married. In terms of education, 59.9% (303 participants) were university-educated or higher, while 34.8% (176 participants) had completed secondary school. Furthermore, 42.7% (216 participants) were students, and 20% (101 participants) were housewives.

**Table 1:** Sociodemographic characteristics of the participants (n = 506) Number (No.), Percentage (%)

Parameter		No.	%
Age (years)	Less than 20	124	24.5
	From 21 to 30	234	46.2
	From 31 to 40	79	15.6
	From 41 to 50	69	13.6
BMI	Underweight	74	14.6
	Healthy	224	44.3
	Overweight	114	22.5
	Obese	86	17.0
	Extremely obese	8	1.6
Educational status	Illiterate/uneducated	1	.2
	Primary	5	1.0
	Middle	21	4.2
	Secondary	176	34.8
	University or more	303	59.9
Nationality	Saudi	410	81.0
	Non-Saudi	96	19.0
Work/profession	Housewife	101	20.0
	Student	216	42.7
	Unemployed	56	11.1
	Office worker	14	2.8
	Educational worker	30	5.9
	Healthcare worker	56	11.1
	Others	33	6.5
Marital status	Single	290	57.3
	Married	208	41.1
	Divorced	5	1.0
	Widow	3	.6

**Figure 1:**
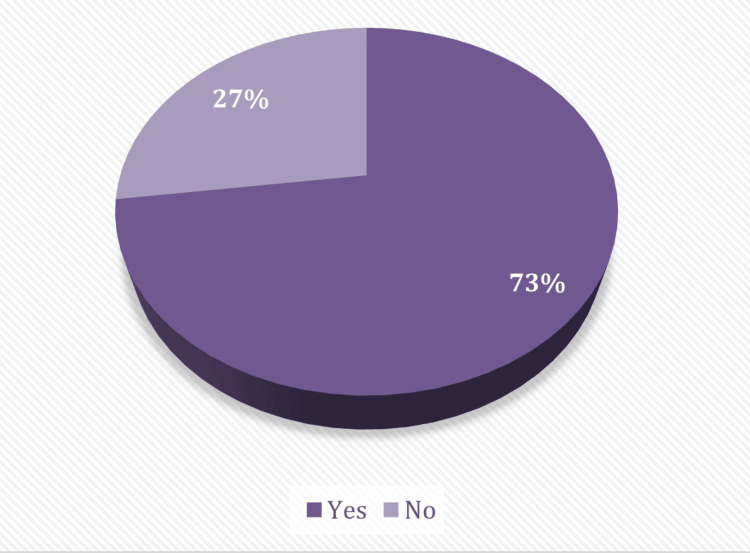
Prevalence of PMS/PMDD among the study participants (n = 506). PMS: premenstrual syndrome, PMDD: premenstrual dysphoric disorder

Figure 1 shows that 369 (72.9%) participants have PMS.

In Table 2, out of the participants, 84.7% (428 participants) reported having children. Among them, 21.3% (108 participants) had five children or more, 21.9% (111 participants) had four children, and 23.5% (119 participants) had two children. Furthermore, 18% (91 participants) disclosed using contraceptives, with 22% (112 participants) using IUDs, 20% (101 participants) utilizing oral tablets, and 38.5% (195 participants) opting for condoms. In terms of menstrual cycle regularity, 71.5% (362 participants) reported having a regular cycle, while 28.5% (144 participants) experienced irregular cycles.

**Table 2:** Number of children and contraceptive use among the study participants (n = 506) Number (No.), Percentage (%)

Parameter		No.	%
Have children	Yes	183	84.7
	No	33	15.3
If you have children, how many children do you have?	1	29	15.8
	2	43	23.5
	3	32	17.5
	4	40	21.9
	5 and over	39	21.3
Use contraceptive	Yes	91	18.0
	No	125	24.7
	Unmarried	290	57.3
If yes, what is the method?	Oral tablets	19	20.9
	Intrauterine device (copper or hormonal)	20	22.0
	Surgical methods (vasectomy or tubal ligation)	9	9.9
	Subcutaneous contraceptive capsules	8	8.8
	Condom	35	38.5
Age at the first period	Less than 13 years old	216	42.7
	13-15 years old	276	54.5
	Over 16 years old	14	2.8
Menstrual cycle regular	Yes	362	71.5
	No	144	28.5

Moving on to PMS symptoms in Table 3, 79.1% (400 participants) indicated mood changes, 59.3% (300 participants) reported experiencing tantrums, 56.7% (287 participants) noted changes in appetite, 47% (238 participants) reported acne, 41.9% (212 participants) experienced back and joint pain, and 43.1% (218 participants) suffered from sleep disorders. When queried about the duration of symptoms, 40.1% (203 participants) mentioned experiencing symptoms over three days, 30.2% (153 participants) over two days, and 7.3% (37 participants) over one day. In addition, 73.9% (374 participants) observed a decrease in work productivity due to symptoms, while 7.9% (40 participants) reported that PMS had a negative impact on their social relationships.

**Table 3:** Symptoms of PMS/PMDD among the study participants (n = 506). Number (No.), Percentage (%), PMS: premenstrual syndrome, PMDD: premenstrual dysphoric disorder

Parameter		No.	%
Symptoms before period (biased data)	Fatigue and extreme tiredness	181	35.8
	Change in mood	400	79.1
	Tantrums	300	59.3
	Poor attention and focus	148	29.2
	Anxiety	186	36.8
	Changes in appetite and cravings for sugar	287	56.7
	Suddenly feeling sad	311	61.5
	Sleep disorders	218	43.1
	Which is insomnia or sleeping a lot	218	43.1
	Acne	238	47.0
	Pain in the back, joints and breasts	212	41.9
	Severe headache	33	6.5
	Bloating and weight gain due to fluid retention in the body	179	35.4
	Confusion	179	35.4
	Social withdrawal	117	23.1
	Extremity sweating	77	15.2
	I do not suffer from any symptoms before the period	22	4.3
	Other	41	8.1
Duration of symptoms	Day	37	7.3
	Two days	153	30.2
	Three days	203	40.1
	Other	113	22.3
Symptoms affect the productivity of day of work and tasks.	Yes	374	73.9
	No	132	26.1
Premenstrual syndrome affect social relationships.	Slightly affected	203	40.1
	Moderately affected	149	29.4
	Severely affected	40	7.9
	Not affected	114	22.5

In Table 4, it is highlighted that 42.1% of the participants (213 individuals) sought treatment to alleviate the symptoms of premenstrual syndrome. Within this group, 72% (153 participants) opted for paracetamol, while 6.1% (13 participants) chose NSAIDs. Moreover, 55.9% (283 participants) shared that the treatment they received helped relieve their symptoms of premenstrual syndrome. In addition, 16.4% (83 participants) engaged in exercise during their menstrual cycle to alleviate symptoms, while 31% (157 participants) focused on consuming healthy foods during this period for symptom relief. Furthermore, 74.5% (377 participants) implemented relaxation techniques during their menstrual cycle to alleviate symptoms of premenstrual syndrome.

**Table 4:** Management of PMS symptoms among the study participants (n = 506). Number (No.), Percentage (%), PMS: premenstrual syndrome

Parameter		No.	%
Receive any treatment to relieve the symptoms of premenstrual syndrome	Yes	213	42.1
	No	293	57.9
If yes, what kind of treatment?	NSAID - non-steroidal anti-inflammatory drugs	21	6.1
	Paracetamol (Panadol)	247	72.0
	Other	75	21.9
Treatment received relieve the symptoms of premenstrual syndrome	Yes	283	55.9
	No	223	44.1
Exercise during the menstrual cycle to relieve symptoms of premenstrual syndrome	Yes	83	16.4
	No	423	83.6
Eat healthy foods at the time of period to relieve the symptoms of premenstrual syndrome	Yes	157	31.0
	No	349	69.0
Relax at the time of the menstrual cycle to relieve the symptoms of premenstrual syndrome	Yes	377	74.5
	No	129	25.5

Table 5 shows a significant association between age and PMS/PMDD, with a higher percentage of individuals aged less than 20 reporting symptoms compared to other age groups. However, no significant association was found between BMI categories and PMS/PMDD. In terms of educational status, the data indicated that participants with a university education or higher were more likely to report PMS/PMDD compared to those with other educational backgrounds. Nationality, work/profession, marital status, and contraceptive use also showed some associations with PMS/PMDD, although not all of these relationships were statistically significant.

**Table 5:** Association between the sociodemographic characters of the participants with PMS/PMDD (n = 506). p < 0.05 is considered significant. PMS: premenstrual syndrome, PMDD: premenstrual dysphoric disorder

		PMS/PMDD		Total (N = 506)	P-value
		Yes	No		
Age	Less than 20	76	48	124	0.001
		20.6%	35.0%	24.5%	
	From 21 to 30	176	58	234	
		47.7%	42.3%	46.2%	
	From 31 to 40	56	23	79	
		15.2%	16.8%	15.6%	
	From 41 to 50	61	8	69	
		16.5%	5.8%	13.6%	
BMI	Underweight	52	22	74	0.929
		14.1%	16.1%	14.6%	
	Healthy	161	63	224	
		43.6%	46.0%	44.3%	
	Overweight	85	29	114	
		23.0%	21.2%	22.5%	
	Obese	65	21	86	
		17.6%	15.3%	17.0%	
	Extremely obese	6	2	8	
		1.6%	1.5%	1.6%	
Educational status	Illiterate/uneducated	0	1	1	0.225
		0.0%	0.7%	0.2%	
	Primary	4	1	5	
		1.1%	0.7%	1.0%	
	Middle	17	4	21	
		4.6%	2.9%	4.2%	
	Secondary	121	55	176	
		32.8%	40.1%	34.8%	
	University or more	227	76	303	
		61.5%	55.5%	59.9%	
Nationality	Saudi	296	114	410	0.445
		80.2%	83.2%	81.0%	
	Non-Saudi	73	23	96	
		19.8%	16.8%	19.0%	
Work/profession	Housewife	81	21	102	0.205
		22.0%	15.3%	20.2%	
	Student	144	72	216	
		39.0%	52.6%	42.7%	
	Unemployed	44	12	56	
		11.9%	8.8%	11.1%	
	Office worker	10	4	14	
		2.7%	2.9%	2.8%	
	Educational worker	22	8	30	
		6.0%	5.8%	5.9%	
	Healthcare worker	41	15	56	
		11.1%	10.9%	11.1%	
	Other	27	5	32	
		7.3%	3.6%	6.3%	
Marital status	Single	200	90	290	0.073
		54.2%	65.7%	57.3%	
	Married	163	45	208	
		44.2%	32.8%	41.1%	
	Divorced	3	2	5	
		0.8%	1.5%	1.0%	
	Widow	3	0	3	
		0.8%	0.0%	0.6%	
Use contraceptives	Yes	61	30	91	0.007
		12.1%	5.9%	18%	
	No	198	217	415	
		39.1%	42.9%	82%	

## Discussion

Premenstrual symptoms can affect any woman of reproductive age, from menarche through menopause. PMS is a common complaint among reproductive-age women. Premenstrual discomfort affects around 70-90% of women of reproductive age in the United States. PMS-like symptoms are reported by roughly one-third of these women. PMDD, the most severe form of PMS, has been reported in 3-8% of these patients [15].

According to our study results, 72.9% of the studied sample have PMS. According to a study conducted in Dammam, KSA, 96.6% of the investigated sample had at least one premenstrual symptom, and 37.5% had a high symptom severity score [16]. PMS afflicted 37.0% of women in Jeddah, Saudi Arabia [17]. Another population-based study found that 91% of individuals reported at least one symptom, 10.3% experienced PMS, and 3.1% matched the criteria for PMDD [18]. Another study conducted in India found that the combined prevalence of PMS and PMDD was 43% and 8%, respectively [19]. Women in the United States experience 481 menstrual cycles during their lives, according to Halbreich et al. When two pregnancies and postpartum periods are factored in, many women have 459 cycles during their reproductive years. Furthermore, women with PMDD in the United States experience an average of 6.4 days of severe symptoms every menstrual cycle, which is nearly equivalent to eight years of debilitating symptoms throughout the menstrual cycle. Examining the evidence reveals that PMS or PMDD can have negative repercussions and functional deterioration over a woman's entire life, making it a substantial health issue [20].

PMS symptoms can be minor, moderate, or severe. Possible symptoms include changes in appetite, weight gain, headaches, nausea, constipation, anxiety, irritability, aggressiveness, tiredness, restlessness, mood swings, and weeping. Other symptoms include back pain, lower back pain, stomach discomfort, and headaches [21]. Irritability, depression, crying/tearfulness, and anxiety are all psychological symptoms of PMS. PMS causes abdominal bloating, breast discomfort, and headaches. In our study, 79.1% of the participants reported mood changes, 59.3% tantrums, 56.7% change in appetite, 47% acne, and 41.9% back and joint pain and 43.1% reported sleep disorders. 

The duration of affective symptoms varies, ranging from a few days to two weeks. Usually, two days prior to menstruation, symptoms peak and become more intense a week before. Drinking alcohol is associated with a marginally elevated risk of PMS. [22]. Thus, recording the patient's alcohol consumption history can help with counseling and symptom relief. In the current study, 40.1% reported three days, 30.2% reported two days, and 7.3% reported one day.

In our study, age was significantly associated with PMS symptoms among participants. Previous studies have found inconsistent connections between sociodemographic variables and PMS/PMDD. PMS/PMDD was shown to be more prevalent among women who were past reproductive age, had lower levels of education, and were unemployed in a previous study [18]. There was no association with advanced age in Takeda et al.'s survey or Potter et al.'s cohort analysis of 2,863 French women, although age dependency could not be examined in several other research since the samples' age ranges were so narrow [23,24]. Potter et al. did not find the same correlation that Cohen et al. had, and their analysis found no link with the work status. 

Women who were jobless or experiencing stress at work had an increased odd ratio for a high degree of common symptoms, in accordance with a cross-sectional population-based Swedish study that looked at relationships between women's overall health and their employment and working environment [25]. These findings are consistent with prior research and support the idea of a negative relationship between perceived symptom severity and amount of control. According to a previous study, adolescents were expected to have a higher prevalence of PMS, accounting for 49.6% of the population [19].

The current investigation discovered a substantial correlation between contraceptive use and PMS/PMDD. It agreed with the conclusions of earlier research examining the connection between OC usage and premenstrual symptoms. Previous research has shown that there may be no advantages for mood symptoms or even that OCs may have a harmful impact on PMS, such as persistently depressed mood, although more recent research linked OCs to benefits, especially for physical premenstrual symptoms. Preparations with drospirenone and third-generation progestogens even showed improvements in psychological symptoms [26-30]. Globally, it was shown that OCs had no impact on the frequency or pattern of menstrual symptoms [31]. As an illustration, Segebladh et al. compared women with and without unfavorable mood effects from COCs and found a substantially greater frequency of mood disorders and PMS in those with mood effects from COCs [32]. The results of this study suggest that the therapeutic effect of COCs, which replicate the ovarian cycle naturally, is little and that a potential weakness may have a bigger impact. The somewhat beneficial impact may be attributed to patients feeling more in control as a result of cycle consistency brought on by COC.

PMS in the mother, one's personal experiences with mental strain, exercise, eating sweet foods, and coffee were all found to be significantly associated with the frequency of premenstrual symptoms in a previous study, but they only accounted for 14% of the variability in the multiple regression model [16]. Another study found that poor physical and mental health were strongly linked to PMS and PMDD. Sociocultural variables tend to influence the occurrence, understanding, and controlling of PMS. Considering the correlation between inadequate physical well-being and notable psychological discomfort, it is reasonable to conclude that women who satisfy the PMDD criteria have a broader underlying susceptibility that should be taken into account in clinical therapy and future research [18].

This study had several limitations, which included the focus on a specific region, potentially limiting the generalizability of the findings. In addition, the study relied on self-reported data from participants, which may introduce bias and impact the accuracy of the results. Furthermore, the absence of a control group for comparison may limit the ability to draw definitive conclusions about the prevalence, pattern, and predictors of PMS and PMDD in the Asir Region.

In terms of future implications, additional research could expand the scope of the study to encompass multiple regions within Saudi Arabia to provide a more comprehensive understanding of PMS and PMDD prevalence, patterns, and predictors across the country. Longitudinal studies could be initiated to monitor changes in the prevalence, pattern, and predictors of PMS and PMDD over time specifically in the Asir Region. Furthermore, intervention studies could be designed to explore effective strategies for managing and treating PMS and PMDD in Saudi Arabia, considering cultural and societal factors that may influence women's experiences and perceptions of these conditions. Such research efforts could contribute to improving the diagnosis, treatment, and overall well-being of individuals affected by PMS and PMDD in the region.

## Conclusions

The prevalence of PMS/PMDD was among the reported figures worldwide. There was a significant association between age and the use of contraceptives with symptoms of PMS/PMDD among the studied sample. Future studies with larger sample sizes are demanded to assess the burden of PMS/PMDD symptoms among Saudi women.
